# Correlation between Accuracy in Computer-Guided Implantology and Peri-Implant Tissue Stability: A Prospective Clinical and Radiological Pilot Study

**DOI:** 10.3390/jcm12155098

**Published:** 2023-08-03

**Authors:** Pier Paolo Poli, Mattia Manfredini, Carlo Maiorana, Federica E. Salina, Mario Beretta

**Affiliations:** 1 Department of Biomedical, Surgical and Dental Sciences, University of Milan, 20122 Milan, Italy; pierpaolo.poli@unimi.it (P.P.P.); carlo.maiorana@unimi.it (C.M.); salinafederica@gmail.com (F.E.S.); mario.beretta@unimi.it (M.B.); 2 Implant Center for Edentulism and Jawbone Atrophies, Maxillofacial Surgery and Dental Unit, Fondazione IRCCS Cà Granda Ospedale Maggiore Policlinico, 20122 Milan, Italy

**Keywords:** computer-guided implantology, dental implants, digital dentistry, peri-implant hard tissue, peri-implant health, peri-implant soft tissue

## Abstract

The present pilot study was designed by hypothesizing a possible correlation between lack of accuracy in implant placement and peri-implant hard and soft tissue health. A total of five patients underwent computer-guided implant surgery and full-arch immediate loading between 2013 and 2014. They subsequently underwent postoperative cone-beam computed tomography (CBCT). After a follow-up of 5 years, all patients were recalled for a clinical-radiographic evaluation of peri-implant health status. The mean linear deviation was 0.5 ± 0.2 mm at the implant’s head and 0.6 ± 0.2 mm at the implant’s apex, while the mean angular deviation of the long axis was 2.8° ± 1.2°. A mean marginal bone loss (MBL) of 1.16 ± 0.94 mm and 2.01 ± 1.76 mm was observed after 1 and 5 years of follow-up, respectively. At 5 years, the mean peri-implant probing depth (PPD) was 4.09 ± 1.44 mm, 66.6% of the evaluated implants showed peri-implant bleeding on probing (BOP), keratinized mucosa (KM) was <2 mm in 48.4% of cases, and mucosal recession (REC) ≥ 1 mm was assessed in 45.4% of the included implants. A negative correlation was observed between bucco-palatal/lingual linear inaccuracy and MBL, PPD, BOP, and KM.

## 1. Introduction

Over the last few years, following the introduction of cone-beam computed tomography (CBCT) and the development of software for computer-guided implant positioning, a significant technological evolution in the fields of implantology and oral rehabilitation has begun. As a matter of fact, increasing evidence advocates for the association of computer-guided implantology and flapless surgical approaches thanks to the superimposition of patients’ clinical features and radiological data. These techniques, after an accurate diagnostic process, allow simplifying the surgical phase while reducing the invasiveness of the operation to a minimum [1]. At the same time, computer-guided workflows yielded favorable results in terms of accuracy, with linear deviations of less than one millimeter and angular deviations of the implant axis of no more than 4–6 degrees [2,3]. Thus, computer-guided implant insertion can be particularly useful in different challenging clinical situations. These include anatomical regions where correct prosthetically driven implant positioning may be hindered by the amount of available bone in atrophied ridges, or by noble structures located in close proximity to the ideal implant site. In addition, patient-related conditions where implants should be inserted with minimal elevation of mucoperiosteal flaps or via a flapless surgical approach can also benefit from computer-guided surgery [4,5]. Accuracy, as mentioned above, is a critical parameter for assessing the reliability of computer-guided implantology. It is described as the deviation between the pre-operative implant’s position planned in the planning software and the position actually obtained in the intra-operative surgical phase [6]. At the same time, the health and stability of peri-implant hard and soft tissues are another key factor in the long-term prognosis of implant-supported rehabilitation [7,8]. It is noteworthy that, to the best of the authors’ knowledge, there is very little evidence currently available that investigates and describes the clinical and radiological relationship between implant health and accuracy with respect to implant positioning using static protocols. This aspect might be of interest in the clinical setting when understanding whether a reduction in the degree of accuracy in terms of linear and angular deviations could have repercussions on the clinical and radiographic variables related to peri-implant hard and soft tissue health years later. In view of the above, the aim of the present study was to evaluate the possible correlation between the accuracy of implant positioning achieved with a computer-guided workflow and the health conditions of peri-implant hard and soft tissues in terms of marginal bone resorption, the amount of keratinized mucosa, peri-implant vestibular and/or lingual/palatal recessions, the depth of peri-implant probing, and the presence of plaque and bleeding at peri-implant probing. The null hypothesis was that there would be no statistically significant correlation between increasing the degrees of inaccuracy between planned and achieved implant position and the worsening of the peri-implant health status assessed clinically and radiographically.

## 2. Materials and Methods

**Study design:** The present study has been designed as a monocentric prospective clinical and radiological pilot study. The research was conducted in accordance with the fundamental principles of the 1975 Helsinki Declaration with respect to clinical analysis involving human subjects, as revised in 2008, and was additionally approved by the local ethics committee of the Fondazione IRCCS Cà Granda Ospedale Maggiore Policlinico (Milan area 2) in relation to digital workflows in implant dentistry (#0002693-U). All patients involved in the present study signed an informed consent for participation.

Patients were enrolled according to specific inclusion criteria: (1) male or female patients aged 18 years or older; (2) total or partial edentulism with terminal dentition at the maxillary and/or mandibular arch; (3) any extraction of residual teeth performed ≥2 months prior to the planning phase; (4) any bone regeneration surgery performed ≥6 months prior to the planning phase; (5) presence, at the time of implant insertion, of an adequate amount of bone (approximately ≥ 6 mm in thickness) evaluated radiographically in order to avoid simultaneous bone augmentation procedures; (6) presence of a quantity of keratinized mucosa ≥ 2 mm circumferentially around the future implant site. The following local exclusion criteria were adopted: (1) local mucosal inflammation and the possible presence of periodontal disease; (2) presence of erosive mucosal disease; (3) presence of bone lesions; (4) history of local radiation therapy; (5) parafunctional patients with bruxism habit; (6) patients with inadequate oral hygiene or otherwise unmotivated for home care. The following systemic exclusion criteria were identified: (1) chronic pathologies requiring the administration of antibiotic prophylaxis (rheumatic disease, bacterial endocarditis, and congenital heart valve anomalies); (2) medical conditions that require the prolonged use of steroid drugs and bisphosphonates; (3) immunocompromised patients with a history of leukocyte deficiency; (4) patients with coagulation disorders; (5) patients with a history of neoplastic diseases that require or have required the use of radio or chemotherapy; (6) patients with a history of renal failure; (7) patients with a history of uncontrolled endocrinopathies; (8) patients with physical and/or mental handicaps that prevent proper and adequate oral hygiene; (9) patients subject to alcohol or drug abuse; (10) patients with HIV; (11) smoking patients (>10 cigarettes/day); (12) presence of conditions or circumstances that could in any way interfere with the proper participation of the patient in the study. Each patient underwent static computer-guided implant surgery according to the 3DIEMME digital workflow (3DIEMME srl, Cantù, Como, Italy) using the Camlog guide system (Camlog Biotechnologies AG, Basel, Switzerland) between 2013 and 2014. Implants (Screw-line Camlog Guide, Promote Plus, Camlog Biotechnologies AG, Basel, Switzerland) were subsequently loaded using immediate-loading protocols with implant-supported screw-retained provisional prostheses relined intraoperatively. The final prosthesis was delivered after 6 months. In addition, each patient underwent follow-up orthopantomography performed 12 months after prosthetic loading. After a follow-up of 5 years, all patients treated with computer-guided implant placement and immediate loading who underwent accuracy evaluation by means of postoperative CBCT scan were recalled for a clinical and radiographic evaluation of the peri-implant health status.

**Figure 1:**
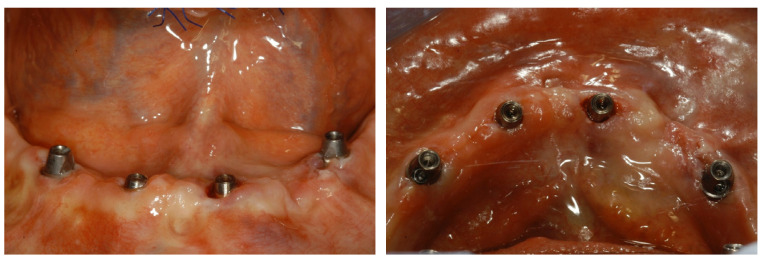
Frontal and occlusal view of peri-implant soft tissues around definitive abutments screwed to the implants.

**Computer-guided workflow:** The same procedures as those described in a previous article were carried out [6]. In brief, a preliminary prosthetic wax-up, corresponding to the exact replica of the final prosthesis accepted by the patient, integrated with aesthetic and functional principles was realized. Then, a radiological stent was fabricated on the basis of the preliminary prosthetic wax-up as a duplication of the final prosthesis. The said stent was equipped with an extraoral radiopaque marker for 3D position tracking, necessary during the subsequent superimposition of the scans. Each subject underwent a CBCT scan of the edentulous jaw while wearing the radiopaque stent in order to integrate the anatomical data with the functional and aesthetic parameters. Subsequently, an optical scan of the prosthesis itself was performed, as required by the digital workflow. The aforesaid scans were imported and matched within the planning software, and the ideal virtual 3D implant position was decided according to the prosthetic design and the jaw’s anatomy by the surgeon, a dental expert in dental prosthesis, and an experienced dental technician in charge of the fabrication of temporary and definitive prostheses. The overlapping was possible thanks to the processing of the data in stereolithographic interface format (STL) acquired from the optical scan superimposed on the data obtained from the CT device using the DICOM format, further using the geometric marker present in both scans. This allowed the simultaneous display of the axial, 3D, panoramic, and transverse images integrated with the prosthetic profile on the computer monitor. Technically, the All-on-4 treatment concept surgical protocol was generally followed [9]. In brief, the two most anterior straight implants followed the bone anatomy in terms of direction. The two posterior implants were inserted just anterior to the foramina or tangent to the maxillary sinus and were tilted distally by approximately 30°, or up to 45° when needed, relative to the occlusal plane. The aim was to reduce, as much as possible, the cantilever’s length and increase the interimplant’s distance, as the posterior implants typically emerged at the second premolar position. Care was taken to avoid conflict between the apices of the anterior and the tilted posterior implants. In the case of thin residual bone crests, implants were tilted slightly palatally/lingually in order to follow the jaw’s anatomy, engage both buccal and palatal/lingual cortical plates, and achieve adequate primary stability. Straight or angled definitive abutments of variable heights were chosen from the library so that prosthetic screw access holes were in occlusal or lingual/palatal locations to allow an acceptable thickness of the prosthesis and to facilitate passive fitting. At this point, the virtual project was transferred into a surgical guide by means of rapid prototyping and stereolithography techniques. All surgical procedures were performed by the same surgeon on an outpatient basis under local anesthesia. The surgical stent was fixed in the correct position using a silicone index, with the guided insertion of surgical pins on the buccal side of the alveolar process according to the virtual plan in order to preserve anatomical structures. The guide allowed the use of calibrated drills, without changing the metal cylinders contained in the stents up to the implant’s insertion. Initially, circular mucosal operculectomy was performed with a surgical mucotome to remove the gingival plug from the implant site, followed by serial osteotomies performed using disposable internally cooled drills, until the planned depth was reached. The implants (Screw-line Camlog Guide, Promote Plus, Camlog Biotechnologies, Basel, Switzerland) were then placed in the desired position according to the manufacturer’s instructions. After the removal of the pins and surgical template, definitive abutments were screwed to the implants (Figure 1).

**Figure 2:**
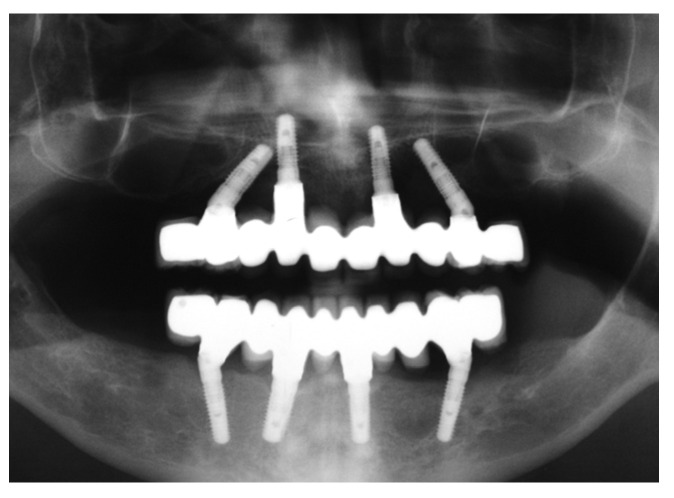
Orthopantomograph obtained after the delivery of the definitive prosthesis.

A temporary screw-retained prosthesis that was relined intraoperatively was finally delivered to the patient. An occlusal check and adjustment were performed to attain optimal distribution of mastication forces. The final rehabilitation was delivered after 6 months, and follow-up orthopantomography was performed (Figure 2).

**Figure 3:**
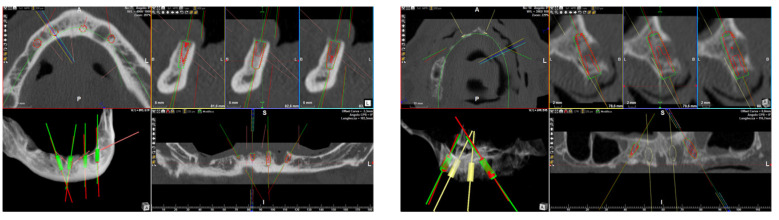
Accuracy evaluation between the planned (red) and real (green) implant positions.

**Accuracy evaluation:** The same procedures as those described in a previous article were carried out [6]. In brief, a postsurgical CBCT scan was conducted with the same apparatus and settings as the preoperative radiological exam. The pre- and post-operative CBCT scans were then overlapped using a specific algorithm in order to compare the virtually planned and actual implant positions and to determine the accuracy level. More in detail, the protocol comprised different phases: (1) extrapolation of the STL files of the bone acquired from the pre-operative CBCT scan via a segmentation process and converting data from DICOM to STL format by means of dedicated software (RealGUIDE 5.0, 3DIEMME, Como, Italy, and Mimics, Materialise, Leuven, Belgium); (2) extrapolation of the STL files of the bone and the final implant positions acquired from the pre-operative CBCT scan via the same segmentation process used above; (3) overlapping of the bone structures retrievable in both pre- and post-operative CBCT scans, which remained unchanged following the surgical procedure (Figure 3). This was an essential aspect as it allowed placing the pre-operative virtual implants planned in the software and the post-operative real implants in the same reference system (GeoMagic Wrap 12, Geomagic Inc., Morrisville, NC, USA). (4) Due to the fact that, in the post-operative CBCT scan, the implants were often surrounded by metal artifacts that yielded low resolutions, each implant was replaced with a high-resolution STL file of the implant retrieved from a digital library available in the planning software. (5) The 3D pre- and post-operative positions of each implant were overlapped, and subsequent calculations of the variables of interest were carried out by means of dedicated software (Rhinoceros 5, Robert McNeel & Associates, Seattle, WA, USA). Three deviation parameters were recorded between each planned and placed fixture: linear deviation (mm) at the implant head and apex and angular deviation (°) of the implant long axis. All measurements were conducted using dedicated software (3Diagnosys, RealGUIDE 5.0, 3DIEMME, Como, Italy).

**Figure 4:**
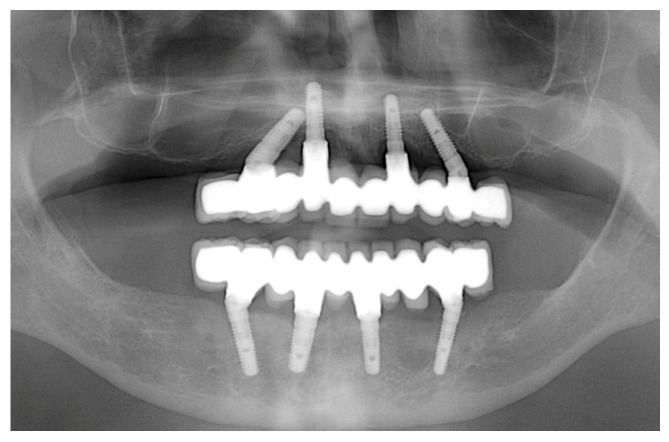
Five-year follow-up orthopantomograph.

**Five-year follow-up examination:** After 5 years, all patients treated with the above-described workflow were recalled for a radiological and clinical evaluation of the peri-implant health status. With respect to the radiographic examination, each patient was prescribed a follow-up orthopantomograph to assess peri-implant marginal bone levels (Figure 4).

**Figure 5:**
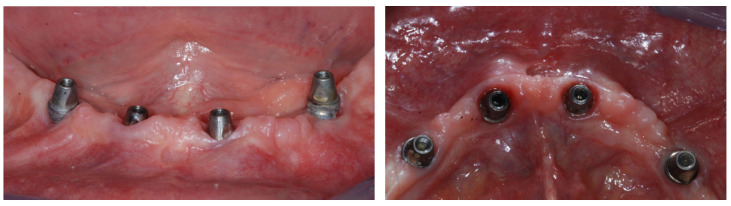
Five-year clinical appearance of the implants and the peri-implant soft tissues at the five-year follow-up recall.

To this end, the orthopantomographs carried out 1 year and 5 years after prosthetic loading were compared digitally to evaluate the variation of peri-implant marginal bone levels throughout the years and determine the peri-implant marginal bone resorption (pi-MBR). In all orthopantomographs, at each implant site, the distance in mm between the implant shoulder and the first visible most coronal bone-to-implant contact reflecting the pi-MBR was measured at 10–15× magnification at both mesial and distal aspects with specific software (Image J, 1.52; U.S. National Institutes of Health, Bethesda, MD, USA). To solve the problem of distortion, the measurements were calibrated on the basis of a known landmark: in this case, the known length of the implant itself. In cases where the peri-implant marginal bone level was more coronal than the implant shoulder as a result of the apico-coronal placement of the implant deeper than the bone crest, a value of “0” was reported regardless of the actual distance between the two references [10]. Considering the clinical examination, definitive implant-supported prostheses were removed, and peri-implant health was evaluated using a millimetric plastic periodontal probe (12-UNC COLORVUE; Hu-Friedy, Chicago, IL, USA) (Figure 5).

In detail, the following clinical parameters were assessed and registered: (a) peri-implant probing depth (PPD) measured at six sites for each implant: buccal, mesio-buccal, disto-buccal, palatal/lingual, mesio-palatal/lingual, and disto-palatal/lingual; (b) peri-implant buccal and palatal/lingual mucosal recession (REC); (c) presence and amount of peri-implant keratinized mucosa (KM); (d) presence of plaque, recorded as a dichotomous variable (YES/NO) by visual inspection; (e) peri-implant bleeding on probing (BOP), recorded as a dichotomous variable (YES/NO) by visual inspection 30 s after a gentle probing of the peri-implant sulcus.

**Statistical analysis:** During the study, data were entered into a spreadsheet (Excel, 15.0.5407.1000; Microsoft Corp., Redmond, WA, USA). Using statistical analysis software (R Statistical Software, v4.0.2; R Core Team 2020), a descriptive analysis of all assessed variables was carried out, and the data were presented as means ± standard deviations. The implant was chosen as the reference statistical unit. As the sample size ranged from 3 to 50, the Shapiro–Wilk test was used to check whether or not the data followed a normal distribution. Depending on the distribution of the data obtained, parametric or non-parametric statistical tests were adopted to evaluate the trend of the study’s variables. To assess whether there were statistically significant correlations between the previously recorded accuracy values and the clinical and radiographic data measured at 12 months and 5 years after prosthetic loading, generalized linear models were constructed using linear and angular deviations as predictors, and pi-MBR, PPD, KM and REC were used as variables. For the dichotomous variables, the implants were dichotomized into two groups, namely with or without plaque and BOP, respectively, and then the values of the variables were quantified in each of the two groups. To see an association between the presence of plaque/BOP and to detect an association between plaque/BOP and the predictors, any significant difference in the distribution of values was quantified by a p-value obtained from a Wilcoxon signed-rank test. For all statistical analyses performed, a *p*-value of <0.05 was considered statistically significant. Statistical analysis and a graphical representation of the results were performed using the R language, version 4.0.2 (R Core Team 2020). The linear models were created using the lm function, and the graphs were generated using the functions of the ggplot2 package (R Core Team 2020).

## 3. Results

The data presented in this pilot study have been prospectively collected from 34 rough-surfaced dental implants placed in five patients (four males and one female) who underwent computer-guided flapless implant placement and immediate loading. Overall, 16 implants were placed in the mandible, and 18 were placed in the maxilla. The implant diameters used were 3.8 mm (27 implants) and 4.3 mm (7 implants), while the lengths used were 11 mm (17 implants), 13 mm (13 implants), and 9 mm (5 implants). Demographic data are reported in Table 1.

All implants achieved adequate primary stability, >35 Ncm, which allowed the immediate loading of a temporary prosthesis [9]; an occlusal check and careful adjustments were performed to attain the optimal distribution of mastication forces in order to not affect the ostiointegration of implants. No implants required bone regenerative procedures. Out of 34 inserted implants, 1 was removed due to peri-implantitis after the delivery of the definitive prosthesis, and it has been replaced with another fixture having the same diameter and length. This latter implant has not been considered in the correlation analysis as it has been placed with a free-hand approach. Of the 34 inserted implants, linear deviations of the implant’s head and apex displacement vector, including the mesio-distal, bucco-lingual/palatal, and corono-apical deviations, and the angular deviation of the long axis are calculated and reported in Table 2.

The mean angular deviation of the long axis was 2.8° ± 1.2°. Considering the 33 implants in function at the latest 5-year recall, a mean pi-MBR of 1.16 ± 0.94 mm was observed at 12 months, whereas a mean pi-MBR of 2.01 ± 1.76 mm was found after 5 years. A mean pi-MBR difference of 0.84 ± 1.99 mm has been noted between 12 months and 5 years, yielding a hypothetical mean annual bone loss of 0.21 ± 0.49 mm. With respect to the clinical variables, a mean PPD of 4.09 ± 1.44 ranging from 1 to 7 mm was measured considering the highest PPD value registered at each of the 33 implant sites among the 6 sites. A total of 22 (66.6%) implants showed BOP; 16 (48.4%) presented with KM < 2 mm; 15 (45.4%) had an REC ≥ 1 mm, ranging from 1 to 4 mm; finally, plaque accumulation was observed in 22 (66.6%) implants, as shown in Table 3.

**Figure 6:**
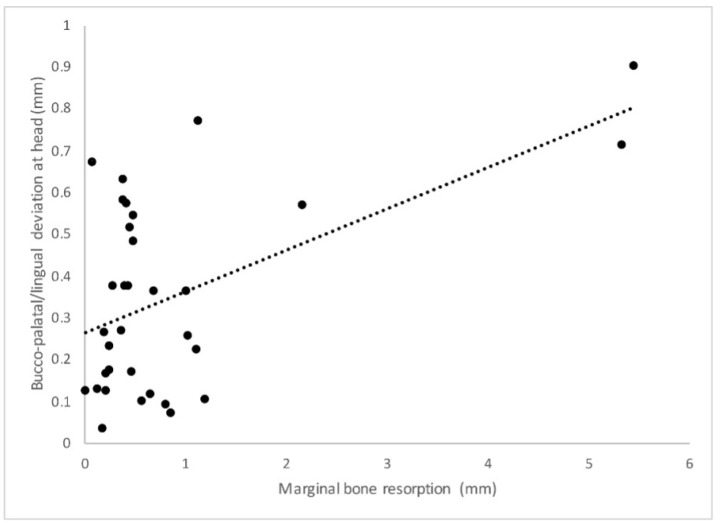
Graph describing the statistically significant correlation (*p* = 0.046) between the implant head deviation in the buccal-lingual/palatal direction and marginal bone resorption.

**Figure 7:**
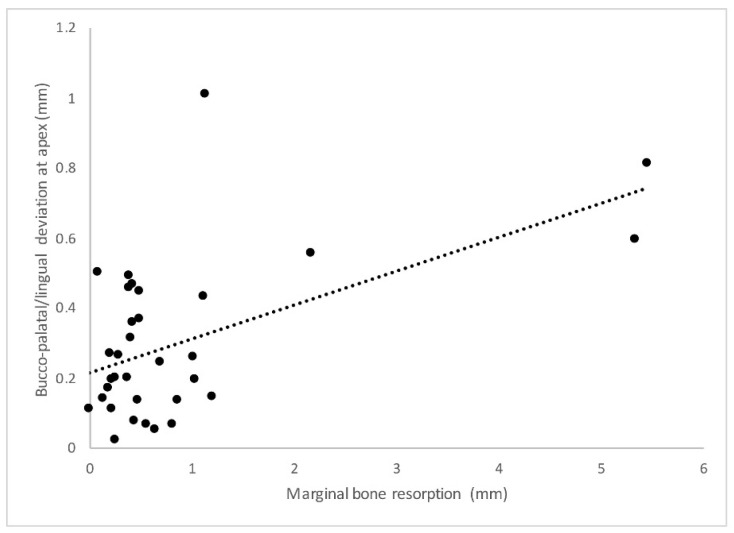
Graph describing the statistically significant correlation (*p* = 0.044) between implant apex deviation in the buccal-lingual/palatal direction and marginal bone resorption.

The results that emerged from the statistical analysis showed a statistically significant correlation between pi-MBR and a bucco-palatal/lingual linear deviation of both the head (Figure 6) and the apex (Figure 7) (*p* = 0.046 and *p* = 0.044, respectively).

Therefore, a tendency toward an increased pi-MBR was found when implants were inserted more buccally. Similarly, a statistically significant correlation between bucco-palatal/lingual inaccuracy and the amount of KM was found for both the head (*p* = 0.029) and apex (*p* = 0.009) so that implants displaced more buccally were more likely to present less keratinized tissue. In a similar fashion, bucco-palatal/lingual inaccuracy at the implant head and apex was also statistically significantly correlated with increased BOP (*p* = 0.031 and *p* = 0.029, respectively) and PPD (*p* = 0.03 and *p* = 0.041, respectively). The remaining linear and angular deviations were not correlated with the radiological and clinical variables in a statistically significant way (*p* > 0.05).

## 4. Discussion

In the present pilot study, the accuracy of implant positioning following flapless computer-guided surgery has been correlated to clinical and radiological variables that commonly define peri-implant tissue health. The aim was to test the null hypothesis that there would be no statistically significant differences between the level of accuracy obtained during the surgical procedure and the peri-implant health status. Results were heterogeneous, as the null hypothesis has been accepted in some cases and rejected in others. In particular, the variables that proved to be statistically significant and correlated with inaccuracy were pi-MBR, KM, BOP, and PPD. In terms of pi-MBR, marginal bone levels measured after 12 months and 5 years from the prosthetic loading were analyzed using orthopantomographs. The data collected from 33 implants were still in function at 5 years and showed a mean pi-MBR of 1.1 mm at 12 months and approximately 2 mm at 5 years. These values are in line with the 2017 World Workshop on the Classification of Periodontal and Peri-Implant Diseases and Conditions definitions of implant health [11] and Albrektsson’s 1986 success criteria [12]. When compared with the latter, the mean value of pi-MBR at 12 months after prosthetic loading observed in the present study is not only within the normal range but is also slightly lower than the mean value reported by Albrektsson et al., which is 1.5 mm [12]. Interestingly, pi-MBR remained rather stable over time, being approximately 2 mm at 5 years, confirming a positive prognosis for the medium-term hard tissue health at implants placed with computer-guided flapless surgery. In this matter, the criteria of implant success described by Albrektsson in 1986 accepted an annual resorption of peri-implant marginal bone of 0.2 mm after the initial remodeling phase [12]. In the present study, the differences in pi-MBR obtained between 12 months and 5 years of function were also analyzed, yielding an overall bone loss of 0.84 mm for 4 years, which was transposed into a 0.21 annual pi-MBR. Although this value approximates the value mentioned above, it remains only hypothetical, as radiological data were not collected on an annual basis. Therefore, any progression or acceleration of the pi-MBR throughout the years cannot be extrapolated from the present data. This constitutes a limitation of the present study. Nonetheless, the results observed herein are consistent with those reported in similar studies. In 2013, Marra and co-workers published a study evaluating pi-MBR after a 3-year follow-up period in 30 fully edentulous patients who had been treated with computer-guided flapless implant surgery [13]. A total of 312 implants were analyzed, and a mean pi-MBR of 1.9 ± 1.3 mm was reported at 3 years. A paper published in 2017 by Lopes et al. evaluated pi-MBR in edentulous patients treated with implant-supported fixed total rehabilitations using the All-on-Four concept by means of computer-guided flapless implant surgery with a 5-year follow-up [14]. Overall, 111 patients were included in the study, and the average marginal bone loss calculated after 5 years was 1.27 mm for tilted implants and 1.34 mm for axial implants. Better results were obtained by Tallarico and co-workers, who published a study comparing pi-MBR values after a 5-year follow-up from prosthetic loading in edentulous patients rehabilitated with implant-supported prostheses undergoing computer-guided flapless surgery [15]. The mean pi-MBR observed after 5 years was 0.87 mm ± 0.40. According to the results of the present study and those found in the pertinent literature, it appears that marginal bone levels obtained after medium-term prosthetic loading following flapless computer-guided workflows are stable and predictable over time. Apart from the pi-MBR values reported to give an overview of the stability of peri-implant hard tissue, data on the accuracy achieved with computer-guided implant surgery were also pivotal in the present study. Accuracy is intended as the matching of the implant’s position planned within the software with that actually obtained in the patient’s mouth. The linear deviations of the implant head and apex measured in the bucco-lingual/palatal, mesio-distal, and corono-apical directions, together with the angular deviation of the implant long axis were considered. It is known that the level of accuracy strongly depends on the reliability and precision of the workflow and methodology used during the planning phase from the diagnosis up to the surgical step. Every aspect must be carefully developed in order to reduce the margin of error in all the steps that characterize the planning phase and the operative phase. An adequate precision and accuracy of all these sequential steps is therefore of paramount importance considering that each error is cumulative and is transferred to the subsequent steps. A review published by Bover-Ramos and colleagues analyzed 34 articles, providing 3033 implants placed with partially and fully guided surgery in vitro (8 studies), in cadaver (4 studies), and in vivo (22 studies) [16]. The data regarding the accuracy of implants placed in patients with fully guided surgical protocols showed an average implant head deviation of 1.00 mm ± 0.08 mm, an average apical deviation of 1.35 mm ± 0.12 mm, and an angular deviation of 3.62° ± 0.29°. No statistically significant differences were also found between the accuracy of implants placed with fully guided surgery in vivo and on cadavers. Marlière and co-workers published a systematic review that included seven studies realized between 2011 and 2016, in which they evaluated the accuracy of implants placed with computer-guided surgery in patients with total rehabilitation [17]. The angular deviation ranged from 1.85° to 8.4°, the implant head deviation fell within a range of 0.71 mm–2.17 mm, and the apical deviation showed an interval of 0.77 mm–2.86 mm. The systematic review with meta-analysis published by Schneider and colleagues included eight studies related to implant placement with computer-guided flapless surgery, in which accuracy was also calculated [2]. Considering in vivo studies only, the mean deviation at the implant head was 1.16 mm, the mean apical deviation was 1.96 mm, and the mean angular deviation was 5.73°. The values found in the present pilot study showed a mean linear deviation of the implant head of 0.57 mm, a mean linear deviation of the implant apex of 0.69 mm, and a mean angular deviation of the long axis of 2.88°. These values were lower when compared to those reported in the systematic reviews mentioned above, meaning that a high level of accuracy was achieved with the workflow described herein. Multiple reasons have been identified: traditionally, intra-oral gutta-percha markers placed inside the radiographic stent were used to integrate the prosthetic plan with the patient’s anatomy in virtual planning. However, in the presence of metal prosthetic restorations, the identification of the radiopaque marker given by the gutta-percha can be challenging. In the present study, an extra-oral radiopaque marker was used, consisting of a well-defined geometric device. A total of 30,000 points were scanned and superimposed during the matching procedure, with greater accuracy in overlapping DICOM data and radiographic stents than traditional protocols.

Additionally, in traditional protocols, two CT scans are usually performed: one of the patient and one of the radiological models. In the present study, the radiological template was scanned using an optical scanner, which provides STL data that are more accurate than DICOM data because they are independent of the Hounsfield unit threshold based on the radiologist-defined gray-level segmentation. This has allowed the surgeon to determine the exact thickness of the soft tissue, resulting in more accurate virtual planning. An optimal level of precision was also achieved via the fixation of the surgical template to the surgical site. The surgical protocol performed involved the insertion of vestibular endosseous pins disposed of in tripod formation, for which their position and depth were guided by dedicated sleeves that were previously established not to interfere with the positions of the implants. In this way, it was possible to avoid movements and deformations of the surgical template caused by the pressures promoted during the preparation of implant sites. Again, in order to achieve a higher level of accuracy in the present protocol, disposable drills were used. In fact, by increasing the cutting capacity, the risk of possible deviations in the osteotomies, caused by excessive wear of the drills, is reduced [6]. The high rate of accuracy found in the present study could explain the medium-term stability of the marginal bone profile evaluated at the 5-year follow-up. On the other hand, the linear deviation of the implant’s head and apex in the bucco-palatal/lingual direction was significantly correlated with the higher values of pi-MBR. A similar trend was also noted for the peri-implant keratinized mucosa. In this case, implants placed more buccally compared to the virtual plan were more prone to develop a contraction of peri-implant hard and soft tissues. A translation of the displacement vector in the vestibular direction could have led to an excessive remodeling of the buccal cortical thickness, with a consequent reduction in the amount of peri-implant keratinized mucosa [18]. In this respect, Monje and colleagues analyzed the critical threshold value of the thickness of the buccal cortical wall to prevent pathological resorption [19]. In particular, an animal study was conducted in which 36 implants were inserted in sites presenting a residual buccal cortical plate < 1.5 mm, while 36 implants were placed in sites that maintained ≥ 1.5 mm of buccal bone. There were no implant failures, but it was interesting to note that implants that were placed too buccally, with a residual buccal bone < 1.5 mm, showed more recession of the buccal mucosa. Interestingly, a recent study published by Romandini and co-workers confirmed that an implant placed too buccally is associated with an almost three times higher risk of presenting peri-implantitis. This was related to the reduced thickness of the residual buccal bone, which is likely to resorb and provoke mucosal recession, with the consequent exposure of the implant surface and increased risk of bacterial colonization [20]. These observations somehow corroborate those reported herein. Although the mucosal recession was not mathematically associated with buccal inaccuracy, a contraction of the peri-implant keratinized mucosa was noted. It can be speculated that with a longer follow-up period, more soft tissue contraction may occur, leading to mucosal recession in accordance with the previous studies. In view of the scientific evidence reported above, the values found in this study regarding the correlation between buccal implant displacement and higher pi-MBR together with a contraction of the keratinized mucosa are interesting. Implants inserted with less accuracy in the bucco/palatal-lingual direction and particularly in a position that is too buccal compared to the initial planning showed greater pi-MBR and less keratinized mucosa. This in turn may lead to an increased risk of developing peri-implant inflammation. Accordingly, the data collected herein supported the fact that the inaccuracy of implant positioning in the bucco/palatal-lingual direction had a statistically significant effect on the presence of peri-implant bleeding on probing. In this respect, Perussolo and colleagues investigated the correlation between reduced peri-implant keratinized mucosa (<2 mm), pi-MBR, plaque, bleeding, and patient discomfort in home oral hygiene procedures [21]. Implants with a reduced amount of keratinized mucosa (<2 mm) were found to be associated with greater pi-MBR, a higher degree of soft tissue inflammation in terms of plaque and bleeding on probing, and finally, greater difficulty in daily home cleaning by the patient. These results are consistent with those obtained in the present work, as pi-MBR, keratinized mucosa, and bleeding on probing were all interconnected and strictly correlated to buccal implant displacement. Although domiciliary compliance was not evaluated in this study, it is likely that buccally placed implants induced a contraction of the keratinized mucosa, leading to more brushing discomfort with consequent plaque accumulation, biofilm-related inflammation, and ultimately bone loss. This favorably complies with a study published by Souza, and co-workers who concluded that implant sites with a band of <2 mm of KM were shown to be more prone to brushing discomfort, plaque accumulation, and peri-implant soft tissue inflammation when compared to implant sites with ≥2 mm of KM [22]. All of this taken together may also explain the significant correlation between inaccuracy and PPD. Indeed, those implants inserted too buccally, that presented with a state of inflammation detected by bleeding on probing, also showed higher values of PPD compared to implants that were placed more accurately. It should be mentioned that, when defining the health of an implant, probing depth values might vary as they are dependent on the peri-implant soft tissue height [23]. Accordingly, a narrative review published by Coli and coworkers aiming at analyzing the correlation between probing depth and peri-implant health, concluded that there is no precise threshold value of PPD that can indicate the presence of disease [24]. In the present work, PPD ranged from 1 to 7 mm, emphasizing the fact that PPD might not be solely correlated to implant health or disease but can also be attributed to other variables not assessed herein. However, the fact that implants placed more buccally were also associated with higher PPD values as a potential consequence of higher inflammation and bone loss is worthy of note. Based on the findings of this study, it is safe to assume that accuracy may play an important role in the stability of peri-implant hard and soft tissues. Clinicians should be aware of the fact that, even in the case of computer-guided implant placement, deviations from the original virtual plan may happen. The more an implant is malpositioned, the higher the risk of compromising the peri-implant health. Thus, as errors are cumulative and may occur during each of the multiple phases of the workflow, all steps have to be carefully managed to minimize inaccuracy. In this respect, an adequate learning curve, the selection of a predictable workflow, and a careful application of the guided surgery protocols are pivotal to achieving successful results in the long term.

It should be stressed at this point that the findings reported in this study should not be overgeneralized due to the small sample enrolled. Indeed, although interesting observations emerged from the data, the lack of external validity remains a limitation of the present research. Therefore, further studies including a larger sample of patients will be needed to validate or deny the correlation between accuracy and the variables analyzed in the present study. These studies should also control for confounding variables such as the bone quality detected in CBCT, primary stability eventually measured with resonance frequency analysis, and implant size and design, which could have partially biased the results reported herein. Finally, it should be taken into consideration that marginal bone loss has been evaluated on orthopantomographs. This does not represent the treatment of choice, as conventional periapical films and digital radiographs showed more accuracy than orthopantomographs in the assessment of peri-implant bone loss [25]. Although periapical radiographs acquired with individualized film holders represent a reliable and reproducible method in multiple assessments of pi-MBR over time in the case of single implants or short-span bridges, this might not be easily applied in the case of full-arch rehabilitations. In such circumstances, especially in patients with resorbed ridges, the ability to position the film holder precisely without distortion of the film itself is reduced. In these cases, a reduced height of the palate following the resorption of the alveolar ridge, or the oral floor and the tongue, may hinder the accommodation of either the film or the film holder in some patients. In the present study, in order to overcome these drawbacks and standardize the measurement method as much as possible, orthopantomographs were used. This strategy is supported by the American Academy of Oral and Maxillofacial Radiology’s position paper, where it is stated that in order to assess dental implant health during the post-implantation period, panoramic radiographs may be indicated for more extensive implant therapy cases, such as those evaluated herein [26].

## 5. Conclusions

In light of the results obtained in the present pilot study, it can be concluded that the accuracy of computer-guided implant placement has an effect on peri-implant hard and soft tissue health. In particular, implants placed more buccally compared to the virtual plan were significantly correlated with higher values of pi-MBR, BOP, and PPD and reduced KM. Thus, reduced accuracy in computer-guided implant placement may adversely affect marginal bone levels and peri-implant soft tissue health.

## Figures and Tables

**Table 1:** Demographic data.

Patient ID	Surgical Site	Age	Sex	Implant ID	Implant Position	Implant Diameter (mm)	Implant Length (mm)
1	Mandible	55	M	1	31	3.8	11
		55	M	2	33	3.8	11
		55	M	3	42	3.8	11
		55	M	4	44	3.8	11
	Maxilla	55	M	5	14	4.3	11
		55	M	6	15	4.3	9
		55	M	7	23	4.3	13
		55	M	8	25	4.3	13
2	Mandible	72	M	9	32	3.8	11
		72	M	10	35	3.8	13
		72	M	11	43	4.3	11
		72	M	12	45	3.8	13
	Maxilla	72	M	13	15	4.3	11
		72	M	14	25	4.3	13
3	Mandible	70	M	15	32	3.8	13
		70	M	16	34	3.8	13
		70	M	17	42	3.8	13
		70	M	18	44	3.8	13
4	Maxilla	62	M	19	12	3.8	11
		62	M	20	14	3.8	11
		62	M	21	16	3.8	11
		62	M	22	22	3.8	11
		62	M	23	24	3.8	11
		62	M	24	26	3.8	11
5	Mandible	42	F	25	32	3.8	13
		42	F	26	35	3.8	13
		42	F	27	42	3.8	13
		42	F	28	45	3.8	13
	Maxilla	42	F	29	12	3.8	9
		42	F	30	14	3.8	11
		42	F	31	16	3.8	11
		42	F	32	22	3.8	9
		42	F	33	24	3.8	9
		42	F	34	26	3.8	9

**Table 2:** Accuracy data.

Patient ID	Implant ID	Linear Deviation (mm)		Angular Deviation (°)
		Head	Apex	
1	1	0.4	0.62	2.12
	2	0.3	1.2	4.94
	3	0.39	0.36	1.85
	4	0.61	0.65	2.33
	5	0.33	0.24	2.69
	6	0.66	0.1	1.11
	7	0.63	0.73	1.82
	8	0.37	0.73	3.22
2	9	0.3	0.37	2.17
	10	0.94	0.94	2.43
	11	0.58	0.67	1.85
	12	0.71	0.66	1.23
	13	0.49	0.65	4.18
	14	1.09	1.1	1.92
3	15	1	0.966	3.4
	16	0.572	0.946	1.79
	17	0.802	0.975	3.44
	18	0.913	0.759	2.46
4	19	0.375	0.849	3.24
	20	0.997	0.633	3.023
	21	0.328	0.9	5.306
	22	0.299	0.321	3.308
	23	0.656	0.674	4.087
	24	0.652	0.722	4.202
5	25	0.585	0.803	0.535
	26	0.844	1.218	2.74
	27	0.613	0.556	2.638
	28	0.75	1	2.282
	29	0.459	0.714	6.189
	30	0.41	0.282	3.166
	31	0.372	0.554	1.931
	32	0.246	0.48	3.035
	33	0.491	0.75	4.961
	34	0.391	0.396	2.492

Mean linear deviations of 0.5 ± 0.2 mm and 0.6 ± 0.2 mm were found for the implant’s head and apex, respectively.

**Table 3:** Data recorded at the 5-year follow-up examination.

Patient ID	Implant ID	Surgical Site	Implant Position	pi-MBR Mesial (mm)	pi-MBR Dital (mm)	Highest PPD (mm)	BOP (+/−)	KM (mm)	REC (mm)	Plaque (+/−)
1	1	Mandible	31	1.03	0.79	5	YES	1	0	YES
	2		33	0.83	1.18	5	YES	0	1	YES
	3		42	1.32	0.73	4	YES	1	1	YES
	4		44	1.22	1.32	5	NO	2	4	NO
	5	Maxilla	14	1.21	0	4	YES	1	1	YES
	6		15	1.32	1.54	4	YES	0.5	0	NO
	7		23	1.98	2.30	4	NO	1	0	YES
	8		25	2.50	2.74	7	YES	0	2	YES
2	9	Mandible	32	0.72	0	2	NO	2	0	NO
	10		35	8.35	7.77	6	NO	2	0	NO
	11		43	2.95	2.64	3	NO	3	0	NO
	12		45	7.16	7.54	6	NO	1	1	YES
	13	Maxilla	15	4.08	4.85	5	NO	4	0	NO
	14		25	3.09	3.70	5	YES	4	0	NO
3	15	Mandible	32	0.00	0.98	1	YES	2	0	YES
	16		34	1.28	1.10	3	NO	2	1	NO
	17		42	0.00	0.00	2	YES	4	0	NO
	18		44	0.75	0.00	2	YES	3	1	YES
4	19	Maxilla	12	2.04	1.74	5	NO	0	2	NO
	20		14	1.67	2.31	4	NO	1	2	NO
	21		16		Implant lost					
	22		22	3.03	3.88	6	YES	0	0	YES
	23		24	2.52	2.76	7	NO	0	0	YES
	24		26	2.40	1.79	6	YES	1	0	YES
5	25	Mandible	32	0.89	1.04	3	YES	3	0	YES
	26		35	0.71	0.00	4	YES	1	2	YES
	27		42	1.63	1.07	3	YES	3	0	YES
	28		45	1.67	1.41	3	YES	3	3	YES
	29	Maxilla	12	1.14	1.74	3	YES	2	0	YES
	30		14	1.96	1.56	4	YES	3	0	YES
	31		16	2.27	2.38	4	YES	2	1	YES
	32		22	3.04	2.82	3	YES	1	0	YES
	33		24	1.09	0.81	3	YES	1	1	YES
	34		26	1.37	1.30	4	YES	2	1	YES

Bleeding on probing (BOP); keratinized mucosa (KM); peri-implant marginal bone loss (pi-MBR); probing depth (PPD); mucosal recession (REC).

## Data Availability

Data are available in the article itself.
